# Characterization of the complete mitochondrial genome of *Sterigmatomyces hyphaenes* (Agaricostilbales: Agaricostilbaceae) and implications for its phylogeny

**DOI:** 10.1080/23802359.2020.1815602

**Published:** 2020-09-04

**Authors:** Maoling Tan, Qiangfeng Wang

**Affiliations:** a College of Food and Biological Engineering, Chengdu University, Chengdu, Sichuan, P.R. China; b Biotechnology and Nuclear Technology Research Institute, Sichuan Academy of Agricultural Sciences, Chengdu, Sichuan, P.R. China

**Keywords:** Yeast, mitochondrial genome, phylogenetic analysis, molecular marker

## Abstract

In this study, the complete mitochondrial genome of *Sterigmatomyces hyphaenes* was sequenced by the next-generation sequencing. The complete mitochondrial genome of *S. hyphaenes* contained 17 protein-coding genes (PCG), 2 ribosomal RNA (rRNA) genes, and 23 transfer RNA (tRNA) genes. The total size of the *S. hyphaenes* mitochondrial genome is 26,198 bp, and the GC content of the mitochondrial genome is 42.08%. Phylogenetic analysis based on the combined mitochondrial gene dataset indicated that the mitochondrial genome of *S. hyphaenes* exhibited a close relationship with that of *Rhodotorula mucilaginosa*.

The genus *Sterigmatomyces* was established by Fell for nonfilamentous, yeast-like fungi, which are characterized by a unique method of cell division (Fell 1966). The yeast cells produce one or more sterigmata, each of which gives rise to a single conidium (Gueho et al. 1990; Messner et al. 1994). Several species have been described in this genus (Sonck 1969; Rodrigues de Miranda 1975). Some species of this genus have excellent salt tolerance, and some species can produce lactosucrose (Lee et al. 2007; Al-Tohamy et al. 2020). Limited morphological characteristics make it difficult to identify or classify *Sterigmatomyces* species accurately only according to morphology (Gueho et al. 1990; Messner et al. 1994). Mitochondrial genome has been widely used in the phylogeny of basidiomycete species (Wang et al. 2020; Li, He et al. 2020). However, up to now, no mitochondrial genome from the genus *Sterigmatomyces* has been published, and the complete mitochondrial genome of *Sterigmatomyces hyphaenes* reported here will promote the understanding of the phylogeny and taxonomy of this fungal group.

The specimen (*S. hyphaenes*) was collected from Sichuan, China (103.26 E; 30.55 N), and was stored in the Culture Collection Center of Chengdu University (No. Asas_ca01). The complete mitochondrial genome of *S. hyphaenes* was sequenced and *de novo* assembled according to previously described methods (Li, Liao et al. 2018; Li, Xiang et al. 2019; Wang et al. 2020). Briefly, we extracted the total genomic DNA of *S. hyphaenes* using a Fungal DNA Kit D3390-00 (Omega Bio-Tek, Norcross, GA). And then we purified the extracted genomic DNA using a Gel Extraction Kit (Omega Bio-Tek, Norcross, GA). The purified DNA was stored in Chengdu University (No. DNA_Asas_ca01). Sequencing libraries were constructed using a NEBNext^®^ Ultra™ II DNA Library Prep Kit (NEB, Beijing, China). Whole genomic sequencing (WGS) of *S. hyphaenes* was conducted using the Illumina HiSeq 2500 Platform (Illumina, SanDiego, CA). We *de novo* assembled the mitochondrial genome of *S. hyphaenes* using SPAdes 3.9.0 (Bankevich et al. 2012; Li, Ren et al. 2020). The complete mitochondrial genome of *S. hyphaenes* was annotated according to the previous described methods (Li, Chen et al. 2018; Li, Wang et al. 2018).

The complete mitochondrial genome of *S. hyphaenes* is 26,198 bp in length, with the base composition as follows: A (28.45%), T (29.48%), G (19.73%), and C (22.35%). The complete mitochondrial genome of *S. hyphaenes* contains 17 protein-coding genes, 2 ribosomal RNA genes (*rns* and *rnl*), and 23 transfer RNA (tRNA) genes. To investigate the phylogenetic status of the mitogenome of *S. hyphaenes*, we constructed a phylogenetic tree for 18 basidiomycete species. *Rhizopogon salebrosus* from the Boletales order was set as the outgroup (Li, Ren et al. 2019a). The phylogenetic tree was constructed using the Bayesian analysis (BI) method based on the combined 14 core protein-coding genes according to previously described methods (Li, Wang, Jin, Chen, Xiong, Li, Liu et al. 2019; Li, Wang, Jin, Chen, Xiong, Li, Zhao et al. 2019; Li, Yang et al. 2020). As shown in the phylogenetic tree (Figure 1), the mitochondrial genome of *S. hyphaenes* exhibited a close relationship with that of *Rhodotorula mucilaginosa* (Gan et al. 2017).

**Figure 1.**
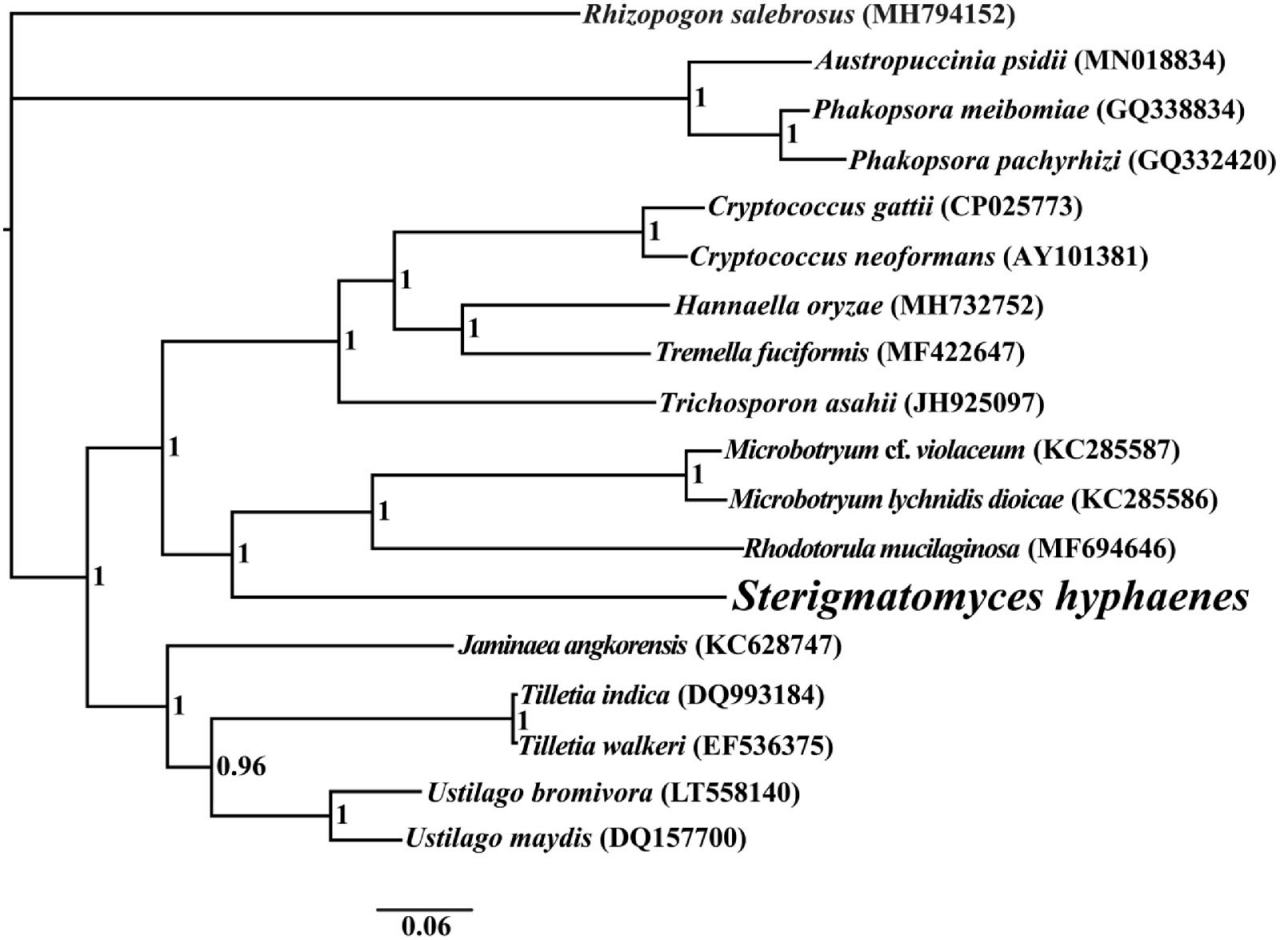
Bayesian phylogenetic analysis of 18 species based on the combined 14 core protein-coding genes. Accession numbers of mitochondrial sequences used in the phylogenetic analysis are listed in brackets after species.

## Data Availability

This mitogenome of *S. hyphaenes* was submitted to GenBank under the accession number of MT755636. (https://www.ncbi.nlm.nih.gov/nuccore/ MT755636).
